# Coordinated roles of SLX4 and MutSβ in DNA repair and the maintenance of genome stability

**DOI:** 10.1080/10409238.2021.1881433

**Published:** 2021-02-17

**Authors:** Sarah J. Young, Stephen C. West

**Affiliations:** DNA Recombination and Repair Laboratory, The Francis Crick Institute, London, UK

**Keywords:** DNA repair, recombination, DNA damage response, crosslinks, telomeres, MUS81, SMX nuclease, XPF

## Abstract

SLX4 provides a molecular scaffold for the assembly of multiple protein complexes required for the maintenance of genome stability. It is involved in the repair of DNA crosslinks, the resolution of recombination intermediates, the response to replication stress and the maintenance of telomere length. To carry out these diverse functions, SLX4 interacts with three structure-selective endonucleases, MUS81-EME1, SLX1 and XPF-ERCC1, as well as the telomere binding proteins TRF2, RTEL1 and SLX4IP. Recently, SLX4 was shown to interact with MutSβ, a heterodimeric protein involved in DNA mismatch repair, trinucleotide repeat instability, crosslink repair and recombination. Importantly, MutSβ promotes the pathogenic expansion of CAG/CTG trinucleotide repeats, which is causative of myotonic dystrophy and Huntington's disease. The colocalization and specific interaction of MutSβ with SLX4, together with their apparently overlapping functions, are suggestive of a common role in reactions that promote DNA maintenance and genome stability. This review will focus on the role of SLX4 in DNA repair, the interplay between MutSβ and SLX4, and detail how they cooperate to promote recombinational repair and DNA crosslink repair. Furthermore, we speculate that MutSβ and SLX4 may provide an alternative cellular mechanism that modulates trinucleotide instability.

## Introduction

The ability of cells to faithfully preserve genetic information is essential for the maintenance of genome stability and the prevention of cancer. Unfortunately, DNA is susceptible to damage from both endogenous (e.g. base misincorporation, base deamination, damage from reactive oxygen radicals, replication stress) and environmental sources (radiation or chemical damage). Therefore, to protect genome integrity, DNA damage is sensed and repaired through a conserved network of proteins and signal cascades, collectively known as the DNA damage response (DDR).

In humans, DNA damage is repaired by a network of lesion-specific repair mechanisms. For example, lesions that minimally distort the DNA helix such as damaged bases (e.g. 8-oxoG) are repaired by base excision repair (BER), whereas bulky adducts that cause local helix distortion, such as pyrimidine dimers, are repaired by nucleotide excision repair (NER). Mismatched bases are repaired by DNA mismatch repair (MMR), and DNA double strand breaks (DSBs) are repaired by non-homologous end joining (NHEJ), single strand annealing (SSA) or homologous recombination (HR). Rare, but damaging, lesions such as interstrand crosslinks (ICLs) can be repaired by DNA glycosylases or the enzymes of the Fanconi anemia pathway. Mutations in DNA repair pathways have been linked to tumourigenesis, in particular breast, ovarian and bowel cancers, as well as neurological and immunological disorders.

## SLX4 plays a role in multiple DNA repair pathways

SLX4 protein is found in a range of eukaryotic species including yeast (Mullen et al. 2001; Fricke and Brill 2003), *C. elegans* (Saito et al. 2009), *D. melanogaster* (Andersen et al. 2009), mouse (Holloway et al. 2011) and humans (Fekairi et al. 2009; Munoz et al. 2009; Svendsen et al. 2009). Human SLX4 is a 200 kDa (1834 amino acid) protein that interacts with and activates three structure-selective endonucleases (SSEs) required for efficient genetic recombination, replication fork restart, telomere maintenance and ICL repair. The importance of SLX4 for genomic stability is highlighted by observations showing that *Slx4^-/-^* mice are born at sub-mendelian ratios and are cancer-prone (Crossan et al. 2011; Holloway et al. 2011; Castor et al. 2013; Hodskinson et al. 2014). Moreover, individuals with biallelic mutations in *SLX4* present with Fanconi anemia (FA), a disease characterized by cancer predisposition and a sensitivity to ICL-inducing agents (Kim et al. 2011; Stoepker et al. 2011). At the cellular level, *SLX4^-/-^* is lethal in chicken DT40 cells, which accumulate in G2-phase with high levels of chromosomal instability (Yamamoto et al. 2011). Mouse or human cells deficient in *SLX4* are sensitive to chemical agents that cause DNA alkylation, ICL-damage and replication stress (Munoz et al. 2009; Svendsen et al. 2009; Crossan et al. 2011; Kim et al. 2013). To date, it has not been possible to produce human *SLX4^-/-^* cancer cell lines by CRISPR-Cas9 gene editing, suggesting that SLX4 may be essential in human tumor cells (Guervilly and Gaillard 2018).

**Figure 1:**
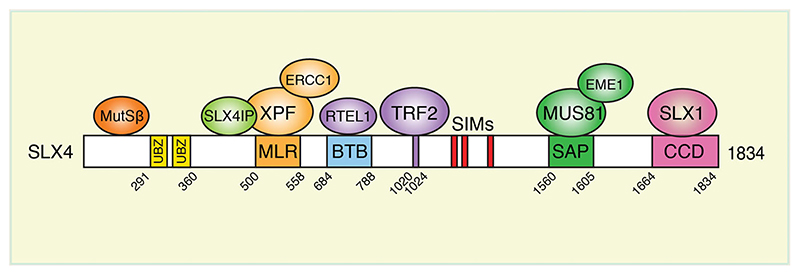
Interactions between SLX4 and MutSβ. Schematic diagram of human SLX4 protein. Selected functional domains and interaction partners are indicated. UBZ, ubiquitin-binding zinc finger domain; MLR, MUS312/MEI-9 interaction like region; BTB, broad complex-tram-track-bric-a-brac domain; SIMs, SUMO-interacting motifs; SAP, SAF-A/B-Acinus and PAIS domain; CCD, coiled coil domain.

SLX4 interacts with three SSEs, SLX1, MUS81-EME1 and XPF-ERCC1, to form the SMX tri-nuclease complex (Fekairi et al. 2009; Munoz et al. 2009; Svendsen et al. 2009; Wyatt et al. 2013; Klein Douwel et al. 2014; Wyatt et al. 2017). SLX4 activates these SSEs and targets them to specific repair contexts by the use of additional interaction partners, post-translational modifications and protein dimerization. For example, SSEs are recruited to telomeres via interactions between SLX4 and the telomere binding proteins TRF2, RTEL1 and SLX4IP (Figure 1). These proteins are required for the regulation of telomere length (Wan et al. 2013; Panier et al. 2019). Moreover, SLX4 binds ubiquitin through its ubiquitin-binding zinc finger (UBZ) domains, and targets the SSEs to ICL damage (Lachaud et al. 2014). SLX4 also binds SUMO through its SUMO-interacting motifs (SIMs), which play a role in targeting SSEs to ICLs, stalled replication forks and telomeres (Gonzalez-Prieto et al. 2015; Guervilly et al. 2015; Ouyang et al. 2015). Human SLX4 exists as a dimer, mediated by its BTB (Bric-a-brac, Tramtrack and Broad complex) domain. This BTB domain is required for telomeric localization and efficient crosslink repair in humans (Guervilly et al. 2015; Yin et al. 2016; Hoogenboom et al. 2019).

## SLX4-interacting nucleases

### SLX1

SLX1 was first discovered in budding yeast as a factor required for cellular survival in the absence of Sgs1 (the yeast homologue of the human BLM helicase) (Mullen et al. 2001). Human SLX1 is a small (28 kDa) protein that contains a GIY-YIG nuclease domain similar to that found in the bacterial NER nuclease UvrC, Type II restriction enzymes and the eukaryotic LEM-3/ANKLE1 nuclease (Dunin-Horkawicz et al. 2006; Brachner et al. 2012; Hong et al. 2018). *In vitro*, SLX1-SLX4 cleaves a variety of branched DNA structures including 5′-flaps, replication forks, splayed arms, recombination intermediates and stem loops. Incisions occur 2–4 nucleotides to the 3′-side of the branchpoint (Fricke and Brill 2003; Coulon et al. 2004; Fekairi et al. 2009; Svendsen et al. 2009; Wyatt et al. 2013).

SLX1 interacts with the C-terminal coiled-coil domain (CCD) of SLX4 (also known as the SLX1 binding domain, or SBD), and SLX1-SLX4^CCD^ exhibits endonuclease activity *in vitro* (Gaur et al. 2015, 2019). In the absence of SLX4, SLX1 exhibits a weak endonuclease activity that is stimulated 500-fold by SLX4 (Fricke and Brill 2003). Structural analyses revealed the molecular basis for this SLX4-dependent activation, such that SLX1 forms a compact stable homodimer *in vitro* that blocks the active site of SLX1. However, in complex with the SLX4^CCD^, the active site of SLX1 becomes accessible (Gaur et al. 2015, 2019).

### XPF-ERCC1

XPF-ERCC1 is best known for the important role it plays in NER, the DNA repair pathway that repairs bulky adducts that arise as a result of exposure to UV radiation. Biallelic mutations in *XPF* are causative of the human disorders Xeroderma pigmentosum (XP) and Cockayne syndrome (CS). XP is characterized by extreme UV sensitivity and cancer predisposition, whereas individuals with CS exhibit developmental and neurological pathologies (Cleaver et al. 2009; Faridounnia et al. 2018). Human XPF is a member of the MUS81/XPF family of 3′-flap endonucleases (Ciccia et al. 2008). In the presence of divalent cations, XPF-ERCC1 cleaves 3′-flaps, bubbles, stem loops and splayed arm DNA structures *in vitro*, 2–8 nucleotides to the 5′-side of the junction (De Laat et al. 1998; Hodskinson et al. 2014). XPF-ERCC1 forms a stable heterodimer mediated through the C-terminus of XPF (De Laat et al. 1998). Structural analysis of truncated human XPF and ERCC1 revealed that the non-catalytic subunit ERCC1 makes direct contact with DNA, indicating that ERCC1 is important for directing XPF activity (Tripsianes et al. 2005). Moreover, DNA-free XPF-ERCC1 was shown to adopt an autoinhibitory conformation that is released upon DNA-junction engagement (Jones et al. 2020). Mutations in XPF that abolish ERCC1 interaction are found in XP patients (De Laat et al. 1998).

A subset of XPF-ERCC1 interacts with SLX1-SLX4 in human cells, mediated by a direct interaction between XPF and the MEI9-interacting region (MLR) of SLX4 (Fekairi et al. 2009; Svendsen et al. 2009; Wyatt et al. 2017). SLX4 stimulates XPF to cleave branched DNA structures *in vitro* including replication forks and ICLs (Munoz et al. 2009; Klein Douwel et al. 2014), consistent with its role in ICL repair. SLX4IP, which is also required for efficient ICL repair, interacts with both SLX4 and XPF-ERCC1 (Zhang et al. 2019). Like *SLX4*, biallelic mutations in *XPF* are causative of FA (Bogliolo et al. 2013). Moreover, expression of SLX4^ΔMLR^ (an XPF-interaction mutant) fails to rescue the sensitivity of *SLX4^-/-^* MEFs to DNA crosslinking agents such as mitomycin C (MMC), and disruption of the SLX4-XPF interaction renders *Xenopus* egg extracts defective for ICL repair (Klein Douwel et al. 2017). Taken together these findings highlight the importance of the SLX4-SLX4IP-XPFERCC1 complex for incision.

### MUS81-EME1

MUS81-EME1 is also a member of the MUS81/XPF family of 3′-flap endonucleases (Ciccia et al. 2008). Purified MUS81-EME1 cleaves 3′-flaps, replication forks and nicked Holliday junctions (HJs) (Boddy et al. 2001; Ciccia et al. 2003; Fricke et al. 2005; Wyatt et al. 2013). MUS81 contains the nuclease motif that catalyzes cleavage, with EME1 playing a regulatory role. MUS81-EME1 is important for various aspects of DNA metabolism in mammalian cells including HJ resolution, ICL repair, replication fork restart and the cleavage of Common Fragile Sites (CFSs) (Svendsen et al. 2009; Kim et al. 2013; Wyatt et al. 2013; Ying et al. 2013; Minocherhomji et al. 2015; Lai et al. 2017). *Mus81^-/-^* mice can be cancer prone, are hypersensitive to ICL-inducing agents and display hallmarks of genomic instability (McPherson et al. 2004; Dendouga et al. 2005).

In higher eukaryotes, the N-terminal helix-hairpinhelix (HhH) domain of MUS81 interacts directly with the SAP domain of SLX4 (Fekairi et al. 2009; Munoz et al. 2009; Svendsen et al. 2009). This interaction appears to have been gained during evolution, as budding yeast Slx4 does not interact directly with Mus81 even though it contains a SAP domain (Schwartz et al. 2012). Interactions between SLX4 and MUS81 enhance the activity of MUS81-EME1 nuclease and broaden its substrate specificity *in vitro* (Wyatt et al. 2017). Activation appears to involve interaction with, and release of, the SLX4-interacting autoinhibitory HhH domain (MUS81^1–86^). Consistent with this, MUS81-EME1 lacking this N-terminal domain exhibits greater nuclease activity toward replication forks than full length MUS81-EME1. As well as modulating the endonuclease activity of MUS81-EME1, SLX4 interaction is also required to target MUS81 to specific genomic locations such as telomeres and CFSs (Naim et al. 2013; Wan et al. 2013).

## Temporal regulation of SLX4 interactions

Genetic, biochemical and structural studies show that SLX4 forms an obligate heterodimer with SLX1, and this interaction is required for the stability and nuclease activity of SLX1 (Castor et al. 2013; Wyatt et al. 2013; Gaur et al. 2015, 2019). SLX1-SLX4 constitutively interacts with a subset of XPF-ERCC1 to form a complex that is stable throughout the cell cycle (known as the SX complex) (Wyatt et al. 2017). In contrast, the interaction of SX with MUS81-EME1 occurs at the G2/M transition, resulting in the formation of an SMX (SLX1-SLX4-MUS81-EME1-XPF-ERCC1) complex that resolves replication/recombination intermediates late in the cell cycle (Wyatt et al. 2013; Duda et al. 2016; Wyatt et al. 2017). Interactions are mediated by mitosis-specific CDK1 and PLK1 phosphorylation events on both EME1 and SLX4. Premature activation of SMX complex formation in human cells during Sphase by inhibition of WEE1 (a negative regulator of CDK1) leads to gross chromosome fragmentation (Duda et al. 2016). Limiting SMX formation to mitosis therefore provides a mechanism to protect replicating DNA from unscheduled cleavage.

## DNA repair pathways that require SLX4

### The resolution of recombination intermediates

In somatic cells, genetic recombination generally occurs between sister chromatids, although a low frequency of events do occur between homologous chromosomes. Recombination leads to the formation of intermediates in which the two interacting sister chromatids or homologous chromosomes are linked by covalent bridges, known as Holliday junctions (Holliday 1964). These arise as products of DSB repair by HR and must be processed to allow efficient chromosome segregation during anaphase (Chan et al. 2018). Cells lacking the ability to process these structures accumulate hallmarks of genome instability and cell death (Wechsler et al. 2011; Sarbajna et al. 2014; Chan et al. 2018).

**Figure 2:**
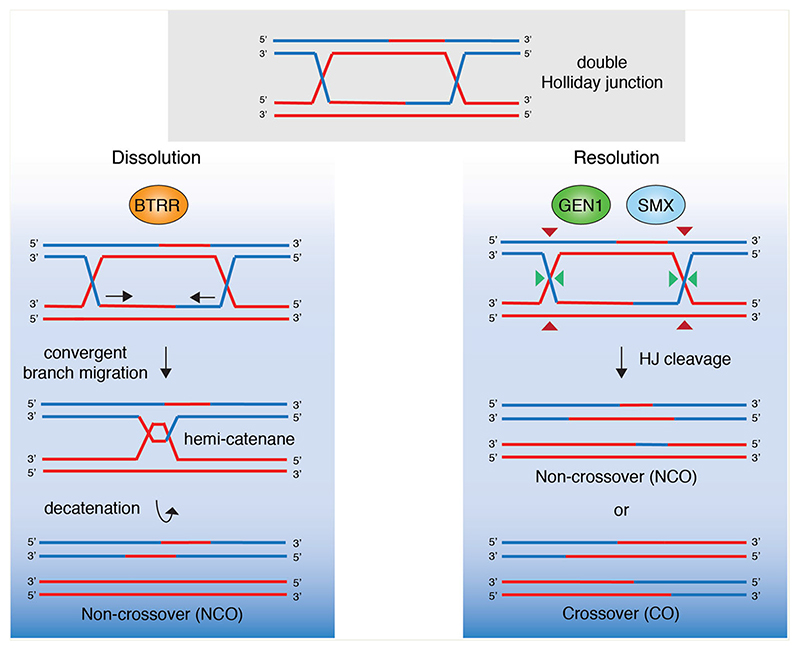
Holliday junction processing in human cells. Covalently linked double Holliday junctions (dHJs), generated during HR are processed by ‘dissolution’ or ‘resolution’. (Left) Dissolution involves the BTRR complex (BLM, Topoisomerase IIIα, RMI1, RMI2). BLM helicase drives convergent branch migration and the resulting hemi-catenane is dissolved by Topoisomerase IIIα. The products of this pathway are exclusively non-crossovers as they do not involve reciprocal exchanges of genetic material between sister chromatids. Right: Holliday junction resolution involves nucleolytic cleavage by the structure-specific endonucleases GEN1 or SMX (SLX1-SLX4-MUS81-EME1-XPF-ERCC1). Resolution gives rise to both non-crossovers (NCOs) and crossovers (COs).

There are two primary pathways by which HJs are resolved (Figure 2), and these involve ‘dissolution’ by the BLM-TopoIIIα-RMI1-RMI2 (BTRR) complex and ‘resolution’ by the SMX complex (Wyatt and West 2014). Dissolution involves helicase/topoisomerasemediated convergent migration of two junctions to form a hemi-catenane that is removed by the topoisomerase, leading exclusively to the formation of noncrossovers (NCOs) (Wu and Hickson 2003; Chen et al. 2014). This prevents loss of heterozygosity (LOH), which can be mutagenic (Wang et al. 2018). Consequently, *BLM^-/-^* cells lacking HJ dissolution display an increased incidence of sister chromatid exchanges (SCEs), which is a hallmark of genomic instability and cancer predisposition (Wu and Hickson 2003; Wechsler et al. 2011). In contrast, resolution occurs through nuclease-mediated nicking followed by religation. Mammalian SMX complex cleaves HJs by a coordinated nick and counter-nick mechanism to form both crossovers (COs) and noncrossovers (NCOs). The formation of COs between sister chromatids can result in LOH. HJ resolution was recently shown to be an essential process in humans as cells lacking HJ resolvases display lagging chromosomes and DNA bridges in mitosis, leading to DNA damage and cell death (Sarbajna et al. 2014; Chan et al. 2018). Interestingly, the COs manifest as SCEs on metaphase chromosome spreads (Wechsler et al. 2011; Castor et al. 2013), which makes it possible to use SCE formation as a readout for the efficiency of HJ cleavage. Loss of SLX4 or MUS81 causes synthetic lethality in *BLM^-/-^* cells that are defective for HJ dissolution (Wechsler et al. 2011; Castor et al. 2013; Wyatt et al. 2013). This lethality is accompanied with chromosome abnormalities and a reduction in SCEs. SLX4 and MUS81 are epistatic supporting the notion that they function in the same pathway of HJ cleavage, one that is independent of a second pathway of resolution mediated by GEN1 endonuclease (Ip et al. 2008; Wechsler et al. 2011). Within the SMX complex, SLX1 and MUS81 are responsible for the initial nick and counter-nick, respectively. XPF-ERCC1 does not appear to be directly involved in cleavage but may stimulate resolution by providing some form of structural stabilization (Wyatt et al. 2017). However, mouse cells expressing a mutant SLX4 lacking the XPF-interaction domain (SLX4^ΔMLR^) do not display defects in SCE formation in *BLM^-/-^* cells, indicating that XPF plays a relatively minor role in HJ resolution (Garner et al. 2013).

### Interstrand crosslink repair

**Figure 3:**
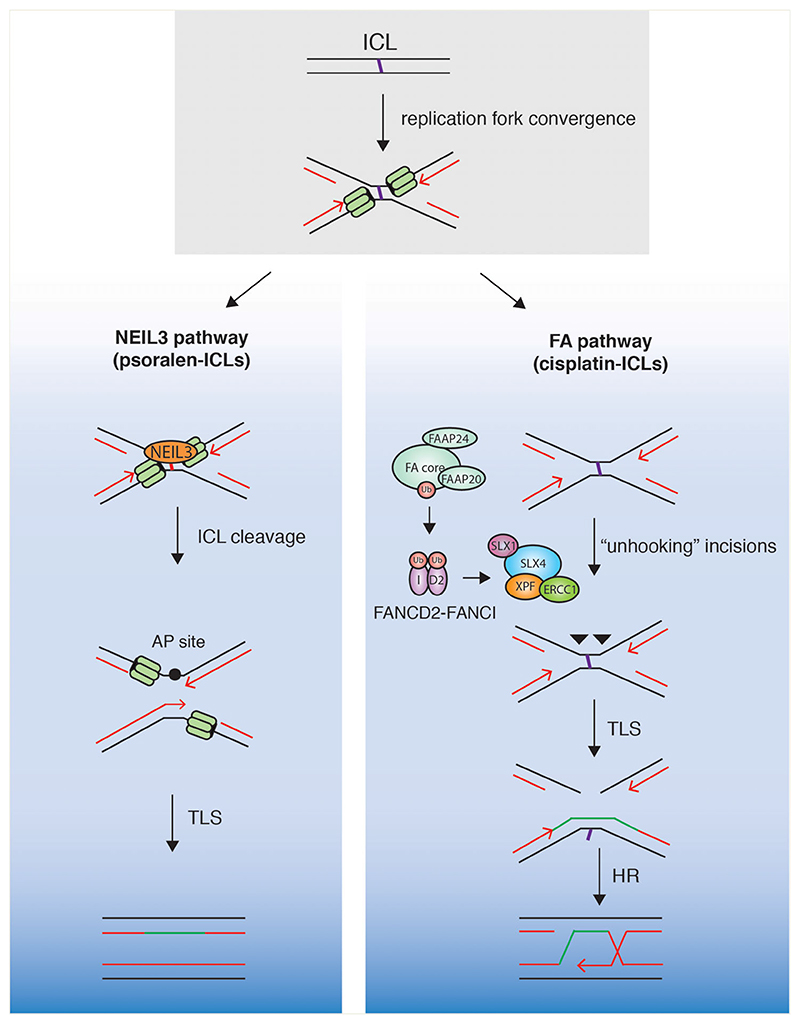
Mechanism of replication-coupled ICL repair. ICLs are repaired during replication by the NEIL3 (left) or Fanconi anemia (right) pathways. Convergent replication forks stall at ICLs. NEIL3 glycosylase is recruited to cleave the ICL and the resulting DNA is repaired by translesion synthesis (TLS). If NEIL3 cleavage fails, the FA pathway repairs the ICL. Activation of the FA core complex and mono-ubiquitination of FANCD2-FANCI, leads to the recruitment of SLX4 with its partner endonuclease XPF-ERCC1. Dual incisions occur on either side of the ICL. The resulting DSB is repaired by TLS and HR.

ICLs are particularly toxic lesions as they prevent strand separation and block the progression of transcription or replication. They are formed as a consequence of endogenous aldehyde metabolism (Garaycoechea et al. 2018) or chemotherapeutic agents (Rycenga and Long 2018). ICLs are primarily repaired by the NEIL3 glycosylase or the Fanconi anemia (FA) pathway (Figure 3), although other pathways of repair have also been reported (Räaschle et al. 2008; Knipscheer et al. 2009; Fu et al. 2011; Klein Douwel et al. 2014; Semlow et al. 2016; Wu et al. 2019; Hodskinson et al. 2020). Pathway choice depends on the structure of the crosslink, with mildly helix distorting psoralen-ICLs repaired by the NEIL3 pathway, whereas profoundly helix distorting cisplatin-ICLs are repaired by the FA pathway. The FA pathway may provide an important backup mechanism when initial NEIL3-mediated repair fails. Individuals with defects in the FA pathway present with the rare genetic disorder FA, which is characterized by developmental defects, progressive bone marrow failure, cancer predisposition and sensitivity to ICL-inducing agents (Niraj et al. 2019).

Mechanistic insights into replication-coupled ICL repair have been provided by *in vitro* reconstitution assays using *Xenopus* egg extracts. In this system, replication forks are seen to stall approximately 20 nucleotides from the crosslink (Räaschle et al. 2008). Fork convergence induces TRAIP (TRAF-interacting protein) to ubiquitinate the CDC45-MCM2-7-GINS (CMG) helicase, and the resulting short ubiquitin chains recruit NEIL3 glycosylase to cleave the crosslink (Semlow et al. 2016; Wu et al. 2019). If cleavage fails, longer ubiquitin chains on CMG promote its unloading from chromatin, leading to replication fork collapse (Wu et al. 2019). Fork collapse then triggers the activation of the ATRmediated DDR, resulting in the phosphorylation and assembly of the multi-protein FA core complex on chromatin (Collis et al. 2008; Kim et al. 2008; Shakeel et al. 2019). The FA core complex initiates the monoubiquitylation of FANCD2-FANCI (Smogorzewska et al. 2007; Shakeel et al. 2019; Tan et al. 2020), and this leads to the recruitment of SLX4 and XPF-ERCC1 which makes dual incisions on one strand at either side of the ICL (Kim et al. 2013; Hodskinson et al. 2014; Klein Douwel et al. 2014, 2017; Hoogenboom et al. 2019). Following ‘unhooking’, the lesion is bypassed by translesion synthesis (TLS), and the resulting DSB is repaired by HR (Hashimoto et al. 2016).

In higher eukaryotes, the SLX1-SLX4-XPF-ERCC1 (SX) complex plays an important role in the initial stages of replication-coupled ICL repair (Klein Douwel et al. 2014, 2017; Hoogenboom et al. 2019). SLX4 contains two N-terminal putative ubiquitin-binding (UBZ) motifs, and UBZ-1 has been shown to bind ubiquitin polymers *in vitro* (Kim et al. 2011; Lachaud et al. 2014). The UBZ domains are required for an interaction with monoubiquitinated FANCD2 and for the recruitment of SLX4 to sites of ICL damage (Yamamoto et al. 2011; Klein Douwel et al. 2014). Cells lacking the SLX4 UBZ domains are hypersensitive to the ICL-inducing agent MMC (Stoepker et al. 2011), highlighting their importance for ICL repair. An N-terminal truncation of mouse SLX4 that contains the UBZ and MLR domains (^mini^SLX4), stimulates ICL cleavage *in vitro*, and is sufficient to rescue the MMC sensitivity of *SLX4^-/-^* MEFs (Hodskinson et al. 2014). Collectively, these studies indicate that ubiquitinated SLX4 targets XPF-ERCC1 to ICLs and stimulates its nuclease activity to perform unhooking. The interdependence of SLX4 and XPF in ICL repair is underpinned by the fact that biallelic mutations in either protein are causative of FA (Stoepker et al. 2011; Bogliolo et al. 2013). Moreover, mutations that abrogate XPFSLX4 interactions are unable to rescue the FA-like phenotype in mice and ICL repair mediated by *Xenopus* egg extracts (Crossan et al. 2011; Kim et al. 2013; Klein Douwel et al. 2017; Hoogenboom et al. 2019).

SLX1 and MUS81 also appear to play roles in ICL repair in higher eukaryotes. For example, *MUS81^-/-^* or *SLX1^-/-^* MEFs are hypersensitive to ICL-inducing agents, albeit to a lesser extent than ERC*C1^-/-^* MEFs (McPherson et al. 2004; Dendouga et al. 2005; Hanada et al. 2006; Hiyama et al. 2006; Castor et al. 2013). Moreover, *Xenopus* egg extracts expressing a SLX4^ΔSAP^ (a MUS81-interaction mutant) or SLX4^DSBD^ (an SLX1-interaction mutant) display only minor perturbations in ICL repair *in vitro* (Klein Douwel et al. 2014; Hoogenboom et al. 2019). Most likely, SLX1 and MUS81 play a role downstream of SLX4-XPF-ERCC1 in ICL repair, in the cleavage of HJs generated during DSB repair by HR.

SLX4IP, a largely uncharacterized SLX4-interaction partner, has also been recently implicated in ICL unhooking by the SX complex. SLX4IP interacts with both SLX4 and XPF-ERCC1, and stabilizes formation of the SX complex. Moreover, *SLX4IP^-/-^* cell lines are sensitive to MMC and show reduced levels of ICL repair (Zhang et al. 2019).

### Replication fork restart

SLX1-SLX4 and MUS81-EME1 are also required for the cleavage and restart of stalled replication forks. Both SLX4 and MUS81 are found at active replication forks and depletion of either protein results in a sensitivity to chemical agents that impede fork progression, such as camptothecin (CPT) or hydroxyurea (HU) (Munoz et al. 2009; Svendsen et al. 2009; Kim et al. 2013; Dungrawala et al. 2015). Replication fork restart can involve the cleavage of a HJ-like reversed fork structure to form a DSB, and it has been shown that SLX4 and MUS81 promote DSB formation and replication fork restart after prolonged stalling by HU treatment (Fugger et al. 2013; Guervilly et al. 2015). Most likely, SLX4 and MUS81 cleave HJ-like reversed forks to promote repair (Wyatt et al. 2013). Consistent with these observations, SLX4^ΔSAP^ and SLX4^ΔSBD^ fail to rescue the CPT sensitivity of *SLX4^-/-^* human cells indicating that SLX4 controls the activity of SLX1 and MUS81 at reversed forks to promote fork restart (Kim et al. 2013). However, unrestrained endonuclease activity at reversed forks is in itself a source of genomic instability and the reversed fork structure is normally protected from SLX4-mediated cleavage by factors such as BRCA1 and BRCA2 (Quinet et al. 2017). Therefore, it is likely that fork cleavage by SLX4-associated endonucleases is a last resort, or pathological response, that allows fork restart.

Interestingly, ATR inhibitors (ATRi) are now commonly used in the clinic for the treatment of cancer, particularly in combination with the replication stress inducing agent HU (Fordham et al. 2018). Mechanistically, ATRi kills cancer cells by causing an accumulation of unrepaired DSBs during replication (Qiu et al. 2018). SLX4 has been shown to be required for ATRi mediated DSB formation and cell death (Couch et al. 2013; Matos et al. 2020), indicating that SLX4 expression levels may be used as a biomarker to identify patients that may respond to ATRi.

### Common fragile site cleavage

CFSs are regions of the genome that tend to display as gaps and breaks in mitotic chromosomes, particularly under conditions of mild replication stress, such as following aphidicolin (APH) treatment. Gap formation is known as CFS ‘expression’. CFSs are frequently associated with breakpoints linked with rearrangements and deletions in cancers (Glover et al. 2017). They tend to be AT-rich and contain long genes with few origins. As such, they are widely regarded to be the last loci to undergo replication, with DNA synthesis at these sites observed into mitosis (Le Tallec et al. 2013; Minocherhomji et al. 2015). Mitotic DNA synthesis (MiDAS) is thought to be a form of break induced replication (BIR) as it is RAD52- and POLD3-dependent (Minocherhomji et al. 2015; Bhowmick et al. 2016). Like BIR, MiDAS differs from conventional replication in that it is conservative and uses the newly synthesized leading strand as a template for lagging strand synthesis, leading to the formation of HJs (Ozer and Hickson 2018). SLX4, MUS81 and XPF all localize to sites of MiDAS and depletion of these proteins in cells treated with APH results in chromosome segregation defects and DNA damage in G1-phase. Presumably, the SMX complex is needed for the cleavage of HJs to allow sister chromatid separation (Naim et al. 2013; Ying et al. 2013; Minocherhomji et al. 2015; Duda et al. 2016). It is thought that SLX4 recruits MUS81 and XPF to these sites and that recruitment requires the SUMO-interacting motifs of SLX4 (SIMs) (Guervilly et al. 2015; Ouyang et al. 2015), suggesting that SUMOylation of SLX4 may play a role in CFS expression by the SMX complex.

### Telomere homeostasis

Mammalian telomeres comprise tandem 5′-TTAGGG-3′ repeats that can range from 10–20 kilobases (kb) in humans, to 50 kb in mice (Shay and Wright 2019). One strand (the G-strand) contains a 3′-ssDNA overhang, that invades the repetitive telomeric DNA to form a telomere-loop (T-loop) (de Lange 2004). T-loop formation protects the chromosome ends from being recognized as a DSB and prevents DDR-mediated repair by NHEJ leading to chromosome fusions. The Shelterin complex, comprising the TRF1, TRF2, RAP1, TIN2, TPP1 and POT1 proteins, is required for T-loop formation and suppression of the DDR at telomeres (Palm and de Lange 2008; Doksani et al. 2013; Lim et al. 2017).

Telomeres shorten during every round of replication and this ultimately leads to replicative senescence as the T-loop can no longer form efficiently (Harley et al. 1990). To prevent senescence, most cancer cells maintain telomere length by reactivating telomerase, a reverse transcriptase that adds telomeric repeats to the ends of chromosomes (Kim et al. 1994). Alternatively, a subset of tumors maintain telomere length without telomerase activity, by an HR-mediated mechanism known as ALT (alternative lengthening of telomeres) (Cesare and Griffith 2004; Wang et al. 2004). Cells using ALT are characterized by the presence of telomeric SCEs (T-SCEs), telomere length heterogeneity and the formation of extrachromosomal telomeric repeat circles (T-circles).

A role for human SLX4 in telomere maintenance was initially indicated by observations showing direct interactions with TRF2 (Fekairi et al. 2009; Munoz et al. 2009; Svendsen et al. 2009). Structural analyses revealed that a leucine residue in SLX4 (SLX4^L1022^) is important for mediating hydrophobic interactions with TRF2 (Wan et al. 2013; Wilson et al. 2013). Consistent with this, expression of SLX4^L1022A^ in U2OS cells (an ALT cell line) resulted in the loss of telomeric SLX4, MUS81 and XPF, supporting the concept that TRF2 recruits SLX4 and its associated SSEs to ALT telomeres. SLX1, MUS81 and XPF have all been shown to be directly involved in telomere processing in ALT cells as they are required for the formation of telomeric sister chromatid exchanges (T-SCEs), in an SLX4-dependent manner (Zeng et al. 2009; Wan et al. 2013). SLX4-associated SSEs are generally considered to be negative regulators of telomere length as cells lacking SLX4 display longer telomeres with increased fragility in both telomerase-positive and ALT mammalian cells (Wilson et al. 2013; Sarkar et al. 2015). Recently, it was shown that SLX4IP maintains telomere by antagonizing BTR complex to favor SMX-dependent T-loop resolution. SLX4 is furthermore inactivated in some ALT-positive tumors and is linked to metastatic recurrence by governing telomere maintenance mechanisms (Panier et al. 2019; Robinson et al. 2020).

The over-processing of telomeres by SLX4-associated SSEs may lead to cellular senescence. Therefore, ALT cells appear to restrict nucleolytic processing through TRF2 and BTR-dependent mechanisms. TRF2 binds to loops formed at ALT telomeres and prevents HJ formation (Schmutz et al. 2017). Moreover, BTR-mediated HJ dissolution antagonizes SLX4-mediated HJ cleavage at telomeres, as it has been shown that depletion of BLM in U2OS cells results in increased T-SCEs and T-circles, and a reduction in telomere length (Sobinoff et al. 2017; Panier et al. 2019).

## SLX4-MutS*β* interaction

Human SLX4 interacts with MSH2 and MSH3 (Svendsen et al. 2009; Gonzalez-Prieto et al. 2015; Zhang et al. 2019; Young et al. 2020). MSH2-MSH3 form a heterodimeric protein known as MutSβ that is required for the repair of heteroduplex loops formed during DNA replication (Fishel 2015). Until recently, however, little was known about SLX4-MutSβ interactions or how they contribute to genomic stability. The remainder of this review will therefore focus on the actions of MutSβ in DNA repair, and in particular how the SLX4 scaffold and MutSβ might cooperate to promote HJ resolution, replication fork maintenance and trinucleotide repeat instability.

## DNA mismatch repair by MutS*α* and MutS*β*

MSH2, MSH3 and MSH6 are eukaryotic homologues of the *E. coli* MMR recognition protein MutS. MSH2 forms an obligate heterodimer with either MSH6 (MutSα) or MSH3 (MutSβ), and the presence of MSH2 is required for the stability of either partner protein (Acharya et al. 1996; Burdova et al. 2015). In human somatic cells, the majority of MSH2 is in complex with MSH6, with approximately 10-fold more MutSα present in HeLa cells than MutSβ (Genschel et al. 1998). Each subunit is composed of five structural domains, including an N-terminal mismatched DNA binding domain (MBD) and a C-terminal ABC ATPase domain (Warren et al. 2007).

DNA mismatch repair is a conserved mechanism that repairs mis-paired nucleotides that arise from DNA damage or replication errors. Although replicative polymerases exert a proofreading function, a subset of nucleotides routinely escape this process, resulting in mismatches (Bebenek and Ziuzia-Graczyk 2018). Polymerases are also prone to slippage during the replication of repetitive sequences. This can result in stretches of mis-paired nucleotide insertions/deletions (IDLs) that form branched heteroduplex DNA structures such as loops or hairpins. Both single nucleotide mismatches and IDLs are substrates for MMR (Levinson and Gutman 1987; Gacy et al. 1995). Defects in the MMR machinery result in a dramatic increase in somatic mutation rates, and are causative of hereditary nonpolyposis colon cancer (HNPCC) (also known as Lynch syndrome), which is characterized by hypermutation and instability of repeat regions known as microsatellites (MSI) (Fishel et al. 1993; Leach et al. 1993; Lynch et al. 1966).

**Figure 4:**
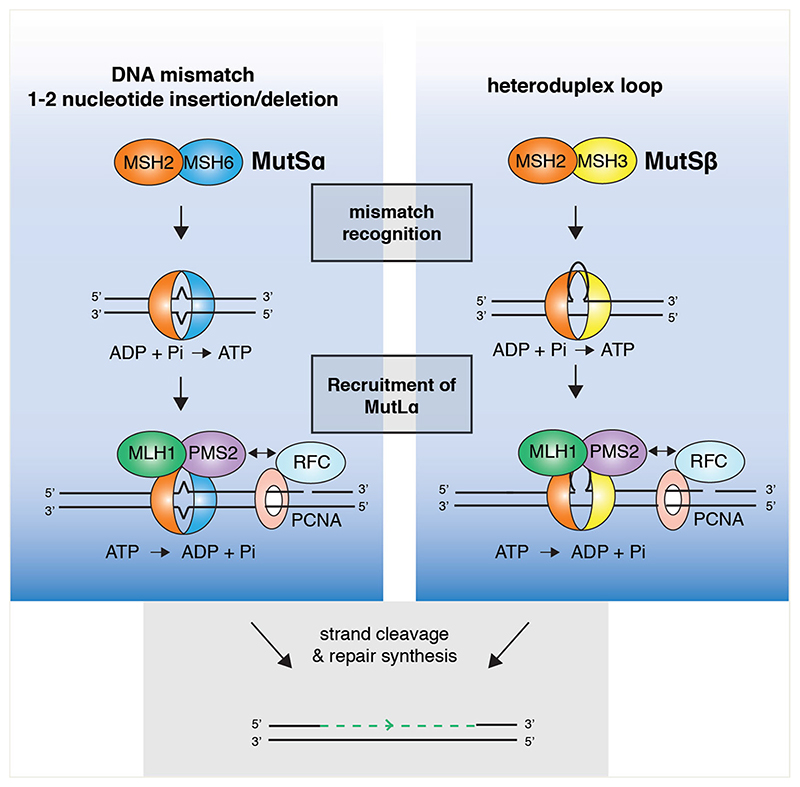
Mechanisms of post-replicative mismatch repair. Single nucleotide mismatches or 1–2 nucleotide insertions/deletions (IDLs) and larger heteroduplex loops are recognized by MutSα and MutSβ, respectively. Mismatch recognition induces ATP binding by MutSα/β which recruits and activates MLH1-PMS2 (MutLα) endonuclease to make single strand nicks. This activity is directed toward the nascent strand containing a preexisting nick through an interaction with PCNA. The mismatch can then be excised by EXO1 and the nascent strand may be re-synthesized by DNA polymerase δ.

MMR comprises four conserved steps (i) mismatch recognition, (ii) cleavage of the nascent strand, (iii) mismatch excision, and (iv) repair synthesis. In higher organisms, mismatches are recognized by MutSα (a heterodimer of MSH2-MSH6) or MutSβ (a heterodimer of MSH2-MSH3) (Drummond et al. 1995; Acharya et al. 1996) (Figure 4). MutSα binds preferentially to single nucleotide mismatches, 1–2 nucleotide IDLs (Gradia et al. 1997, 1999; Warren et al. 2007), damaged bases such as O^6^-methylguanine, and cisplatin adducts *in vitro* (Alani 1996; Duckett et al. 1996; Alani et al. 1997). In contrast, MutSβ exhibits a low affinity for mismatches and single nucleotide insertions, but binds to heteroduplex loops with high affinity (Acharya et al. 1996; Genschel et al. 1998; Wilson et al. 1999; Young et al. 2020). MutSβ also binds (CAG)_13_ repeat hairpins (Owen et al. 2005, 2009; Young et al. 2020), branched DNA structures (Surtees and Alani 2006) and ICLs generated by psoralen or cisplatin (Zhao et al. 2009; Zhu and Lippard 2009). Mechanistic insights into how MutSβ binds a wider range of structures than MutSα was provided by the crystal structures of MutSα and MutSβ in complex with DNA (Warren et al. 2007; Gupta et al. 2011). MutSα interacts with a single G/T mismatch using a conserved phenylalanine residue in the mispair binding domain (MBD) of MSH6, whereas MutSβ interacts with an IDL using a conserved Lys-Tyr motif in the MBD of MSH3. In both cases, MSH2 makes nonspecific contacts with the sugar-phosphate DNA backbone. Whereas MutSα interacts with the base of the mismatch, MutSβ interacts with the phosphate groups in the heteroduplex DNA. This allows MutSβ to have a more flexible DNA binding pocket that is able to accommodate heteroduplex DNA with greater variety of bending angles than MutSα. A chimera of *S. cerevisiae* MutSα with the MBD of MSH3 recognizes IDLs in a manner similar to that shown by MutSβ, highlighting the importance of the MBD in mediating the differential substrate specificities (Shell et al. 2007).

Mismatch recognition causes ATP binding, and leads to the recruitment of MutLα (a heterodimer composed of MLH1-PMS2) (Gu et al. 1998; Gradia et al. 1999; Wilson et al. 1999; Dufner et al. 2000; Mukherjee and Feig 2009) (Figure 4). MutLα then makes a 5′-nick specifically in the strand containing the mismatch. It is thought to target this strand through an interaction with PCNA, which is loaded on DNA in a specific orientation on the nascent strand containing a preexisting nick (Genschel and Modrich 2003; Kadyrov et al. 2006; Pluciennik et al. 2010). *In vitro* reconstitution studies have shown that the mismatch is then excised by the 5′–3′ exonuclease activity of EXO1, and DNA polymerase δ promotes repair synthesis (Genschel and Modrich 2003; Constantin et al. 2005; Zhang et al. 2005).

ATP-binding by MutSα and MutSβ is a critical step in MMR (Figure 4). Mutations in the ATPase domains of MSH2, MSH3 or MSH6, that render them defective in ATP binding, results in MMR deficiency in *S. cerevisiae* (Graham et al. 2018; Kumar et al. 2013), and mutations in the ATP-binding region of *MSH2* are causative of HNPCC in humans (Lutzen et al. 2008; Drost et al. 2013). When ADP-bound, MutSα binds DNA with a high affinity. However, ATP-binding reduces its affinity for DNA (Gradia et al. 1999; Wilson et al. 1999), leading to the formation of a highly processive sliding clamp that promotes the recruitment of MutLα to facilitate repair (Erie and Weninger 2014).

MutSα and MutSβ are required for the recruitment of MutLα (or MutLβ/MutLγ) to complete MMR. MutSα forms a complex with MutLα in an ATP-dependent manner, and the latent endonuclease activity of MutLα is ATP- and MutSα-dependent (Blackwell et al. 1998; Kadyrov et al. 2006). MutSα interacts with the N-terminus of MLH1 and mutations that abolish this interaction are MMR-deficient and cancer-associated (Iaccarino et al. 2000; Plotz et al. 2006). Taken together, these results indicate that the ATP-dependent sliding clamp conformation of MutSα is required for MutLα interaction. As MutSβ may also form a sliding clamp in the presence of ATP, it is assumed that MutSβ acts in a similar manner to MutSα.

## MutS*α* and MutS*β* in homologous recombination

Studies in mouse and human cells indicate that MutSβ plays a role in the early stages of HR. For example, MSH2, MSH3 and MSH6 are all rapidly recruited to sites of IR-induced DSBs in human cells (Hong et al. 2008). Also, radiation treatment of *MSH2^-/-^* or *MSH3^-/-^* MEFs results in the persistence of unrepaired DSBs (indicated by γH2AX foci) and a reduction of HR-mediated repair (indicated by persistent RAD51 foci). This is accompanied with chromosome breaks and decreased cellular survival (Franchitto et al. 2003; van Oers et al. 2014).

MutSα and MutSβ also play a role in the later stages of HR. Early studies showed that budding yeast MutSα binds to a variety of recombination and repair intermediates *in vitro* (Marsischky et al. 1999; Surtees and Alani 2006). Moreover, siRNA depletion of MSH2 in human U2OS cells results in a decrease in T-SCE formation, indicative of a defect in HJ resolution at ALT telomeres (Martinez et al. 2017). Human MutSα also interacts with BLM and helps promote HJ dissolution (Yang et al. 2004). Recently, it was shown that human MutSβ binds HJs with a high affinity and stimulates their resolution by SLX1-SLX4 or the SMX trinuclease (Young et al. 2020). Efficient HJ resolution was dependent on direct interactions between MutSβ and SLX4. Consistent with the biochemical studies, cells defective for *MSH3* exhibited reduced SCE formation and an increased frequency of homologous recombination ultra-fine bridges (HR-UFBs), characteristic of a defect in the resolution of recombination intermediates. In addition, *GEN1*k/o cells depleted for MSH3 exhibited increased fragile site UFB (FS-UFB) formation, indicating that the MutSβ-SMX complex plays a dual role in the resolution of both recombination and late replication intermediates. Stimulation of HJ resolution by SMX was not observed with MutSα, and there was no observed increase in HR-UFB or FS-UFB formation in MSH6-depleted cells.

MutSβ is also thought to play a role in the removal of a 3′-non-homologous tail during single-strand annealing (SSA). This process is important for the repair of DSBs that form between direct repeats. Repair involves the annealing of the repeat sequences on either side of the DSB causing a deletion of the intervening sequences, in a reaction that is RAD52 dependent (Van Dyck et al. 2001). MutSβ plays an essential role in the removal of the non-homologous tails that are generated during the annealing reaction. For example, *S. cerevisiae* MutSβ binds 3′-flaps and is recruited to sites of DSBs in a Rad52-dependent manner (Surtees and Alani 2006). MutSβ facilitates the removal of these tails by interacting with and recruiting the yeast homologue of XPF-ERCC1 (Rad1-Rad10) (Paques and Haber 1997). Although it is currently unclear whether MutSβ and XPF-ERCC1 play similar roles in SSA in humans there are several indications that this may the case: (i) human RAD52 stimulates the cleavage of 3′-flaps by XPF-ERCC1 *in vitro* (Motycka et al. 2004), (ii) ERCC1 interacts with both MSH2 and RAD52 (Lan et al. 2004), and (iii) MutSβ is rapidly recruited to the sites of DSBs in human cells (Hong et al. 2008).

## MutS*α* and MutS*β* in ICL repair

MutSβ interacts with psoralen-induced ICLs in DNA and is required for their efficient repair in cell-free extracts (Zhang et al. 2002; Wu et al. 2005; Zhao et al. 2009). Human cells lacking MutSβ are sensitive to ICLs produced by cisplatin, psoralen and MMC (Zhao et al. 2009; Takahashi et al. 2011; Williams et al. 2011; Park et al. 2013; Sawant et al. 2015). MSH2 and MSH3 interact with SLX4 and XPF-ERCC1 (Lan et al. 2004; Svendsen et al. 2009; Young et al. 2020), possibly within the context of the SMX trinuclease complex, so it is tempting to speculate that MutSβ may play a role in lesion unhooking or subsequent HR-mediated repair. In contrast, MutSα, which fails to interact with SLX4, is not required for ICL repair and cells lacking MSH6 are resistant to cisplatin treatment and promote efficient cisplatin-induced ICL repair (Sawant et al. 2015).

## MutS*β* promotes trinucleotide repeat instability

MutSβ plays a critical role in promoting the pathogenic instability of genomic loci that contain trinucleotide repeats (TNRs) in both dividing and post-mitotic mammalian cells. TNRs are tandem arrays of three nucleotides that are found in exons, introns and 5′- and 3′- untranslated regions (UTRs) of genes throughout the genome. Expansion of these repeat regions is causative of more than 30 human degenerative diseases, including Huntington’s disease (HD) (McDonald et al. 1993), Myotonic dystrophy type 1 (DM1) (Mahadevan et al. 1992), Fragile X syndrome (FRAX) (Verkerk et al. 1991) and Amyotrophic Lateral Sclerosis (ALS) (Pulst et al. 1996). Most individuals possess short repeat tracts that are typically nonpathogenic and retain a stable copy number. However, a subset of individuals carry alleles with longer than average repeat regions (pre-mutation allele). Although these are non-pathogenic to the carrier, they undergo copy number changes both in somatic tissue and over successive generations in offspring. Once the repeat number expands beyond a certain threshold they are deemed pathogenic as they substantially alter the expression of the affected gene (Iyer et al. 2015). Exactly how the TNRs are pathogenic is dependent on the genomic location of the repeat region and its nucleotide composition. For example, HD, characterized by uncontrolled motor movements and cognitive dysfunction, is caused by expansion of CAG repeats from 40 (pre-mutation) to 100+ (pathogenic) in the huntingtin gene (*HTT*) (McDonald et al. 1993). This results in a toxic polyglutamate tract in the HTT protein, which is prone to aggregation and accumulation. On the other hand, DM1, characterized by progressive muscle weakening and loss, is caused by expansion of a CTG repeat tract in the 3′-UTR of the *DMPK* gene (Santoro et al. 2017). CTG expansion is thought to affect RNA splicing, protein production and chromatin structure.

MutSβ plays a critical role in the instability of CAG/CTG repeat tracts (CAG repeats on one strand and CTG on the complementary strand) that are causative of HD and DM1. For example, *Msh2^-/-^* knockout results in a stabilization of 110–120 (CAG) repeats in the *HTT* gene in HD mice, and a shift toward contractions of long (CTG) repeats in DM1 mice (Manley et al. 1999; Savouret et al. 2003) Moreover, *Msh3^-/-^* cells exhibit the stabilization of long (CAG) tracts and (CTG) tracts in HD and DM1 mice respectively, and a later onset of disease phenotype. Interestingly, the same stabilization was not observed for *Msh6^-/-^* mice, indicating that this effect is specific to MutSβ, not MutSα (Dragileva et al. 2009). These observations have been expanded upon in human cells, with CRISPR-Cas9 mediated knockout of *MSH3* resulting in the stabilization of (CAG) repeats in human astrocytes (Keogh et al. 2017). Moreover, loss of MSH2 in induced pluripotent stem (iPS) cells derived from DM1 patients leads to the attenuation of CTG expansion (Du et al. 2013). Consistent with these findings, the levels of instability in HD and DM1 patient cells during differentiation correlate with the expression of MMR proteins (Seriola et al. 2011). Taken together these studies illustrate that MutSβ plays a key role in CAG/CTG instability in replicating cells.

Trinucleotide repeats form extra-helical loops/hairpins during replication that result from DNA polymerase slippage, or other processes that involve DNA strand separation such as transcription and repair. Consistent with this, short oligonucleotides containing as few as 6–10 (CAG) or (CTG) repeats form stable hairpin loops *in vitro* (Gacy et al. 1995). Hairpins have been detected in DM1 patient tissues, with a frequency that positively correlates with the instability observed in different tissues (Axford et al. 2013). MutSβ binds specifically to oligonucleotides containing (CAG) or (CTG) repeats, with a comparable affinity as that observed with small MMR-proficient IDLs (Owen et al. 2005; Tian et al. 2009; Pluciennik et al. 2013).

**Figure 5:**
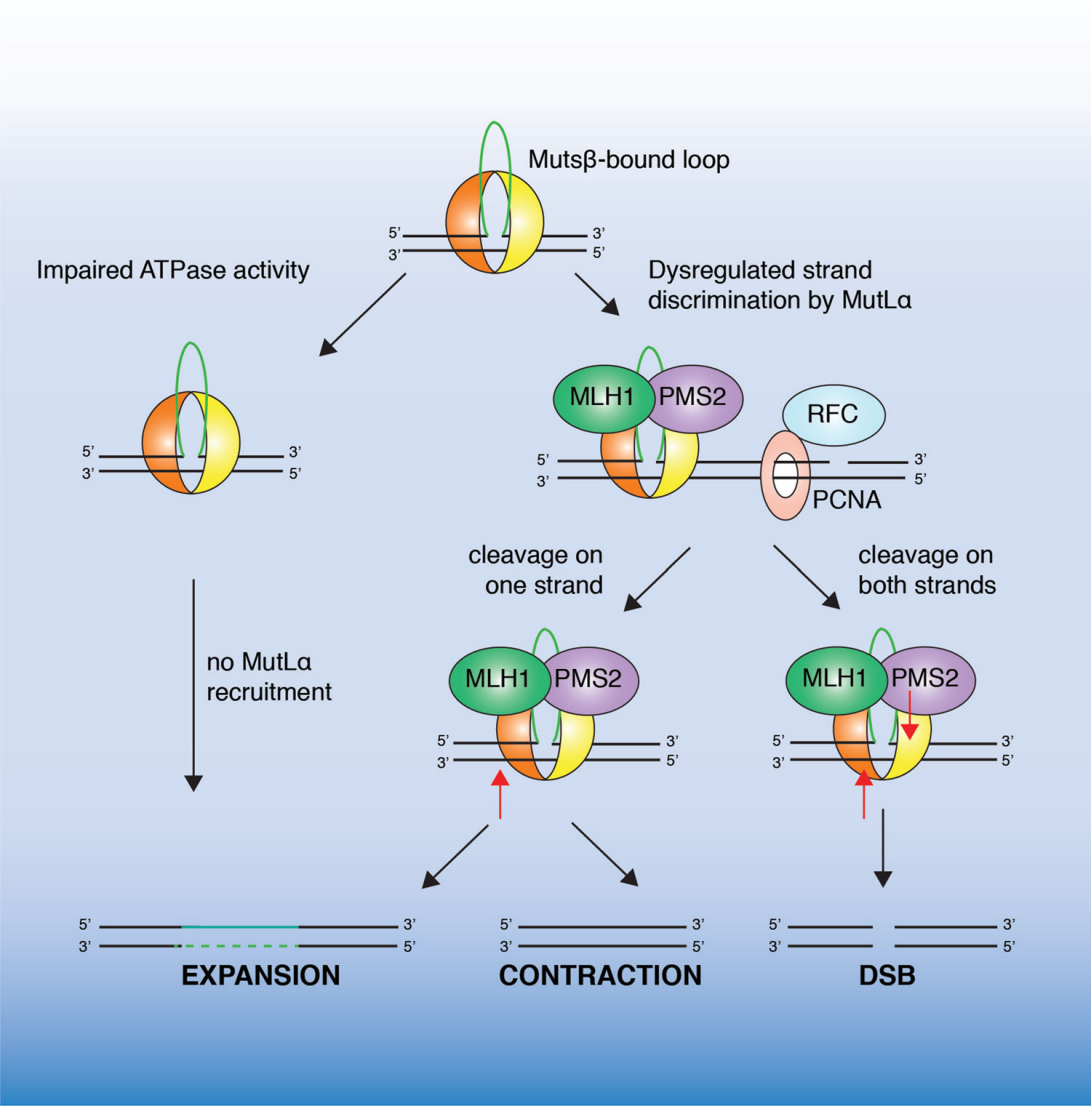
Proposed mechanisms of MutSβ-mediated trinucleotide repeat instability. Impaired MutSβ ATP-binding and sliding clamp formation would prevent loop cleavage by MutLα resulting in expansion. Alternatively, improper PCNA loading may result in dysregulated strand discrimination by MutLα and cleavage on either strand (indicated by a red arrow). This would result in tract expansion, contraction or DSB formation. (see colour version of this figure at www.tandfonline.com/ibmg)

One model for how erroneous MMR may induce TNR instability (Figure 5), the dysregulation of strand discrimination model, dictates that in contrast to canonical post-replicative MMR, where MutLα is directed by PCNA to specifically cleave the nascent strand, PCNA is loaded in either orientation on TNRcontaining DNA (Pluciennik et al. 2010). This would lead to the cleavage of either strand and result in tract instability following repair synthesis. Consistent with this, PCNA can be loaded in either orientation on closed circular DNA containing 1–3 (CAG)_n_ repeats *in vitro*, causing DNA cleavage without strand bias. This model accounts for a role of both MutSβ and MutLα in TNR instability and explains why instability is observed in post-mitotic cells lacking replisomecoupled strand-specific loading of PCNA. A lack of strand-discrimination may also result in the formation of DSBs that are repaired by error-prone recombinational processes such as BIR, resulting in expansions or contractions. Consistent with this model, recombinational repair has been observed to drive large-scale expansions of CAG/CTG repeat tracts in yeast and mammalian cells (Napierala et al. 2002; Kim et al. 2017).

Although MutLα plays a key role in MMR, and is the most abundant MutL complex in the cell, in many model systems it is thought to play only a limited role in trinucleotide repeat expansion. For example, recent studies utilizing a mouse model of Fragile-X related disorders, show that expansion is dependent on the nuclease activity of MutLγ (a heterodimer of MLH1-MLH3), rather than MutLα (MLH1-PMS2) (Hayward et al. 2020). Importantly, DNA cleavage by MutLγ, in contrast to MutLα, does not depend on PCNA loading (Pluciennik et al. 2013; Kadyrova et al. 2020), providing support for a dysregulation of strand discrimination model of TNR instability.

## Interplay between MutS*β* and SLX4 in multiple repair pathways

**Figure 6:**
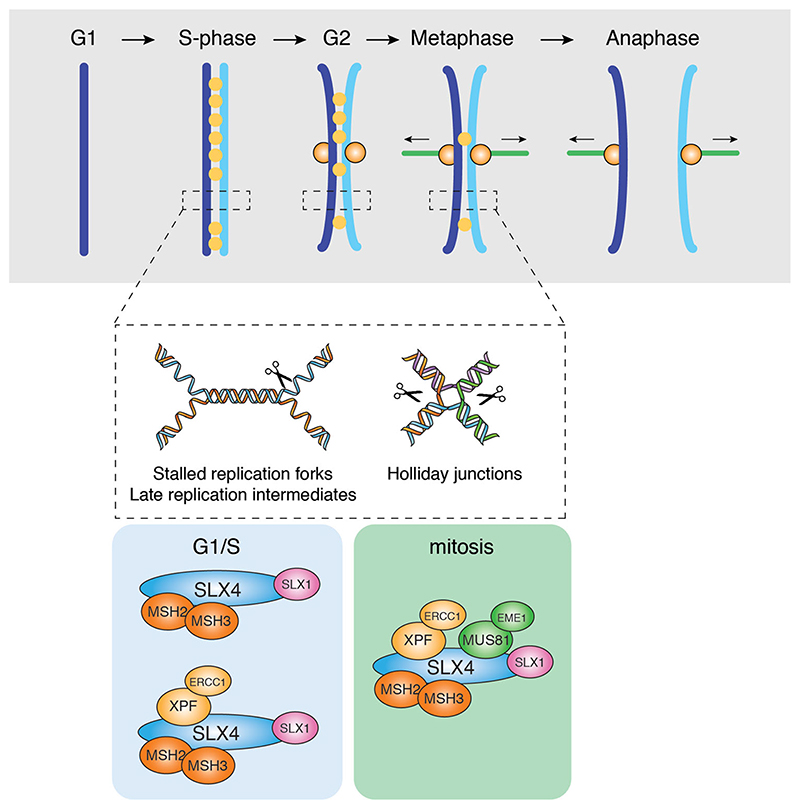
Model of the cooperation between MutSβ and SLX4 complexes in the cleavage of replication and recombination intermediates. During S-phase and G2, MutSβ stimulates the cleavage of replication and HR intermediates by SLX1-SLX4 and/or SLX1SLX4-XPF-ERCC1 (SX) complexes. During mitosis, MutSβ stimulates the cleavage of late replication and HR intermediates by the SMX complex to allow efficient sister chromatid separation in anaphase.

The demonstration of interactions between MutSβ and SLX4 (Svendsen et al. 2009; Guervilly et al. 2015; Zhang et al. 2019; Young et al. 2020), together with observations showing that MutSβ stimulates the nuclease activities of SLX1-SLX4 and SMX trinuclease on recombination intermediates and (CAG) hairpin loops, suggests that MutSβ is an important component of the SLX4 repair complex (Young et al. 2020). The results indicate that a fraction of cellular MutSβ interacts with SLX4 both in S-phase and mitosis, in a manner similar to that observed with SLX4 and XPF-ERCC1 (Figure 6). Several complexes can be envisaged: MutSβ-SLX1-SLX4 and MutSβ-SLX1-SLX4-XPF-ERCC1 in S-phase cells, and the eight subunit MutSβ-SMX complex in mitosis. As a key component of these complexes, MutSβ may help facilitate the targeting of SLX4 and its associated endonucleases to a variety of branched DNA structures, raising the possibility that these proteins play coordinated roles in homologous recombination and trinucleotide repeat instability.

Human MutSβ binds HJs *in vitro* with an efficiency similar to that observed with loop and hairpin structures. Moreover, MutSβ stimulates HJ cleavage by SLX1-SLX4 and the SMX trinuclease complex, regardless of whether it is ADP- or ATP-bound (Young et al. 2020). This contrasts with the way in which MutSβ activates MutLα endonuclease during MMR, which requires MutSβ to be ATP-bound (Kadyrov et al. 2006). MutSβ binds to HJ structures with an affinity higher than that observed with MutSα. In the presence of Mg^2+^, the HJ adopts a stacked X-shaped structure (Duckett et al. 1988), and the difference in binding affinity may be due to the ability of the DNA binding pocket of MutSβ to accommodate a wide range of DNA structures with different bending angles (Gupta et al. 2011). The DNA binding pocket of MutSα on the other hand, is less flexible, and can only accommodate single nucleotide mismatches or 1–2 nucleotide insertions (Warren et al. 2007).

Loss of MutSβ, or components of the SMX trinuclease, leads to the accumulation of HR-UFBs that link sister chromatids during anaphase (Chan et al. 2018; Young et al. 2020). Given that SMX cleaves residual replication and recombination intermediates that persist into mitosis, to allow efficient sister chromatid separation and prevent DNA damage (Naim et al. 2013; Wyatt et al. 2017; Chan et al. 2018), these observations indicate that HJ binding by MutSβ facilitates the recruitment of SMX for UFB cleavage (Figure 6). Similarly, during the repair of ICLs by the FA pathway, we suggest that MutSβ-SLX4 recruits XPF-ERCC1 to perform unhooking incisions around the crosslink, allowing for subsequent repair by HR. SLX4 is thought to be recruited to the ICL by monoubiquitinated FANCD2 (Lachaud et al. 2014). In this regard, it is interesting that MSH2 facilitates the efficient mono-ubiquitination and chromatin loading of FANCD2, and cells lacking MutSβ are sensitive to the ICL-inducing agents MMC, psoralen and cisplatin (Zhao et al. 2009; Williams et al. 2011). Moreover, human MSH2 has itself been shown to interact with XPF (Lan et al. 2004). Taken together, these findings support a role for SLX4-MutSβ complexes in the initial stages of ICL-repair by the FA pathway, in lesion recognition and/or unhooking.

In the case of telomeres, SLX4 is recruited to ALT telomeres by an interaction with TRF2, where it negatively regulates telomere length by counteracting SLX4IP and BLM activity (Wan et al. 2013; Wilson et al. 2013; Sobinoff et al. 2017; Panier et al. 2019). Once targeted, SLX1-SLX4 cleaves recombination intermediate structures at T-loops resulting in the loss of telomeric repeats in the form of T-circles. Interestingly, *MSH2^-/-^* MEFs display an increase in chromosome end-to-end fusions, and depletion of MSH2 in human U2OS cells (an ALT cell line) results in decreased T-SCEs and Tcircles, hallmarks of defects in ALT (Martinez et al. 2017).

The incision of heteroduplex (CA)_4_ loops, (CAG)_13_ hairpins, and poly-T stem loops by human SLX1-SLX4 and SMX *in vitro*, indicates that these nucleases have a propensity for cleaving a diverse range of hairpins that might arise at AT-rich regions and repetitive sequences. Common Fragile Sites are often found at late-replicating AT-rich regions and depletion of MUS81 or XPF in cells treated with low doses of aphidicolin, to induce mild replication stress, leads to mitotic defects, DNA damage, and increased gaps and breaks (Naim et al. 2013; Ying et al. 2013; Minocherhomji et al. 2015; Duda et al. 2016). Given that MutSβ binds tightly to loops and hairpins, we suggest that MutSβ-SMX complexes are likely to play a coordinated role in the processing of late replication intermediates that arise at CFSs in mitosis.

**Figure 7:**
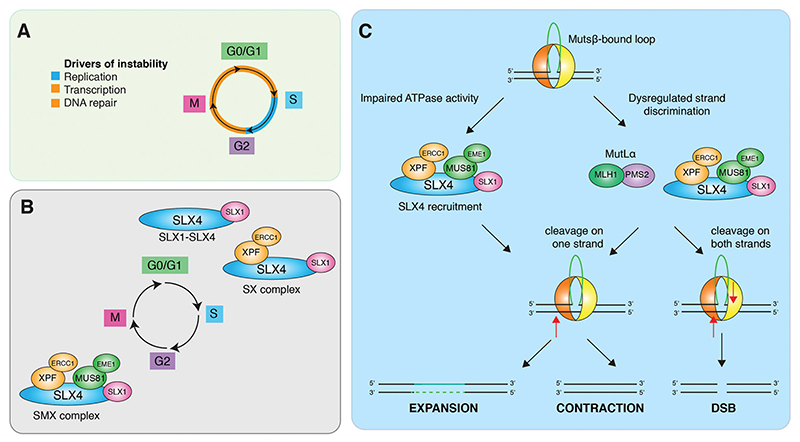
Proposed model for the role of SLX4 in trinucleotide repeat instability. A. Mechanisms that drive trinucleotide repeat instability through the cell cycle. B. Temporal regulation of SLX4-endonuclease complex formation. C. Hypothetical model for the involvement of SLX4 in trinucleotide repeat instability. SLX4-endonuclease complexes are recruited to trinucleotide repeat loops/hairpins by MutSβ where they cleave without strand bias leading to expansions, contractions and DSBs.

Small heteroduplex loops are efficiently repaired by MMR during DNA replication in reactions involving MutSβ and MutLα (Fishel 2015). However, the interaction of MutSβ with SLX4 raises the possibility that loop cleavage by SLX1-SLX4 may provide an alternative, or backup, MMR pathway, especially since SLX4 localizes at active replication forks in human cells (Dungrawala et al. 2015). Trinucleotide repeats also form loops or hairpin structures, and the processing of these branched structures is known to induce the pathogenic expansion of repeat tracts. We therefore speculate that MutSβ-SLX4 complexes could provide an alternative mechanism for trinucleotide repeat instability. Trinucleotide repeat tracts exhibit instability both in replicating and post-mitotic (G0) cell types (Gonitel et al. 2008; Gomes-Pereira et al. 2014), as a result of DNA transcription, repair and/or replication (Figure 7(A)). In replicating cells, TNR instability may be mediated by SLX1-SLX4 in G1, S and G2 phases, and by the SMX complex in late G2/mitosis (Figure 7(B)). It has been shown that MutSβ stimulates (CAG)_13_ cleavage by SLX1-SLX4 in the presence of ATP, but not ADP. This is similar to that observed during canonical replicationcoupled MMR, in which a MutSα sliding clamp is required for the recruitment and activation of MutLα (Blackwell et al. 1998; Kadyrov et al. 2006). We therefore suggest that SLX1-SLX4 or SMX complex, like MutLα, may be regulated by ATP-bound MutSβ. *In vitro*, SLX4 complexes cleave (CAG)_13_ DNAs on both strands to generate a variety of different products including flaps and DSBs (Young et al. 2020). This indiscriminate cleavage of (CAG)_13_ DNAs, indicates that MutSβ-SLX4-complexes may drive repeat instability by a mechanism analogous to that of canonical TNR instability leading to both expansions and contractions (Figure 7(C)).

In conclusion, we suggest that MutSβ-SLX4- complexes are likely to play fundamental roles in multiple aspects of DNA metabolism. While it is becoming clear that MutSβ cooperates with SLX4 in the resolution of HR intermediates and that the complex acts upon heteroduplex loop structures that arise during TNR instability, it is also possible that MutSβ-SMX complexes play a significantly broader role in DNA replication, ICL repair and telomere homeostasis. Indeed, our present knowledge may only be scratching the surface of the importance of SLX4-MutSβ interactions in the maintenance of genomic stability.
